# MMP1-1607 polymorphism increases the risk for periapical lesion development through the upregulation MMP-1 expression in association with pro-inflammatory milieu elements

**DOI:** 10.1590/1678-775720160112

**Published:** 2016

**Authors:** Ana Paula Favaro TROMBONE, Franco CAVALLA, Elcia Maria Varize SILVEIRA, Camile Bermejo ANDREO, Carolina Favaro FRANCISCONI, Angélica Cristina FONSECA, Ariadne LETRA, Renato Menezes SILVA, Gustavo Pompermaier GARLET

**Affiliations:** 1 - Universidade do Sagrado Coração, Departamento de Ciências Biológicas e da Saúde, Bauru, SP, Brasil.; 2 - Universidade de São Paulo, Faculdade de Odontologia de Bauru, Departamento de Ciências Biológicas, Bauru, SP, Brasil.; 3 - Universidad de Chile, Facultad de Odontología, Departamento de Odontología Conservadora, Santiago, Chile.; 4 - University of Texas Health Science Center at Houston, School of Dentistry, Department of Endodontics, Houston, USA.

**Keywords:** Periapical diseases, Periapical granuloma, Matrix metalloproteinases, Polymorphism, Genetic, Inflammation, Cytokines

## Abstract

Increased matrix metalloproteinases (MMPs) activity is a hallmark of periapical granulomas. However, the factors underlying the MMPs expression modulation in healthy and diseased periapical tissues remains to be determined.

**Objective:**

In this study, we evaluated the association between the MMP1-1607 polymorphism (rs1799750) and pro-inflammatory milieu elements with MMP-1 mRNA levels *in vivo*.

**Material and Methods:**

MMP1-1607 SNP and the mRNA levels of MMP-1, TNF-a, IFN-g, IL-17A, IL-21, IL-10, IL-4, IL-9, and FOXp3 were determined via RealTimePCR in DNA/RNA samples from patients presenting periapical granulomas (N=111, for both genotyping and expression analysis) and control subjects (N=214 for genotyping and N=26 for expression analysis). The Shapiro-Wilk, Fisher, Pearson, Chi-square ordinal least squares regression tests were used for data analysis (p<0.05 was considered statistically significant).

**Results:**

The MMP1-1607 1G/2G and 1G/2G+2G/2G genotypes were significantly more prevalent in the patients than in controls, comprising a risk factor for periapical lesions development. MMP-1 mRNA levels were higher in periapical lesions than in healthy periodontal ligament samples, as well as higher in active than in inactive lesions. The polymorphic allele 2G carriers presented a significantly higher MMP-1 mRNA expression when compared with the 1G/1G genotype group. The ordered logistic regression demonstrated a significant correlation between the genetic polymorphism and the expression levels of MMP-1. Additionally, the pro- and anti-inflammatory cytokines IL-17A, IFN-g, TNF-a, IL-21, IL-10, IL-9, and IL-4 were significant as complementary explanatory variables of MMP-1 expression.

**Conclusion:**

The MMP1-1607 SNP was identified as a risk factor for periapical lesions development, possibly due to its association with increased MMP-1 mRNA levels in periapical lesions. The MMP-1 expression is also under the control of the inflammatory milieu elements, being the cytokines TNF-a, IL-21, IL-17A, and IFN-g associated with increased MMP-1 levels in periapical lesions, while IL-10, IL-9, or IL-4 presented an inverse association.

## INTRODUCTION

Periapical lesions are characterized by the destruction of periapical tissues as a consequence of the local host response, which is triggered by bacterial infection of pulpal and endodontic environment^18,30^. Among multiple elements involved in host inflammatory immune responses, cytokines are critical determinants of lesions outcome acting as major modulators of the main pathways responsible for tissue destruction^13,18^. While the RANK-RANKL-OPG system, the main responsible for osteoclastogenesis control, is supposed to be one determinant of periapical lesions activity, the balance between MMPs (matrix metalloproteinases, a family of zinc- and calcium-dependent proteases) and TIMPs (the endogenous tissue inhibitors of metalloproteinases) is supposed to determine the outcome of the protease-mediated catabolic response that contributes to the degradation of soft and mineralized tissues surrounding the root apex^18,19,26,34,^.

In physiological circumstances, MMPs play a role in degradation and remodeling of both extracellular and bone matrix proteins. However, the increase in the expression and/or activation of MMPs without a parallel increase of TIMPs to counteract catabolic proteolysis mediate numerous pathological processes, including the periapical and periodontal diseases^6,23,31^. Indeed, the high matrix proteinase activity is a hallmark of periapical granulomas, and is supposed to derive from a combined action of multiple MMPs such as MMP-1, MMP-2, MMP-8, MMP-9, and MMP-13^1,28^.

Within the MMPs found to be overexpressed in inflamed periapex, MMP-1 is supposed to actively contribute to lesion development by mediating inflammatory cell infiltration and tissue degradation, since MMP-1 is the main proteolytic enzyme that targets types I and III collagen fibers, the periapical tissue matrix most abundant components. Indeed, while MMP-1 levels are relatively low in healthy periapical tissues, its levels are upregulated in human and experimental periapical lesions^12,20^. It is important to mention that a high individual variation in the levels of MMP-1 was described in periapical lesions^29^. Therefore, since modifications in MMP-1 levels may directly influence the development of periapical lesions, the identification of the factors that determine the control of MMP-1 expression in periapical environment may comprise important information to predict and prevent lesions development as well to develop therapeutic strategies to limit lesions progression.

Variations in the promoter region of MMP1 gene account for heritable differences in MMP-1 expression, which can consequently influence the susceptibility to certain pathologies^4,29,32^. A specific single nucleotide polymorphism (SNP) at -1607 position of MMP1 gene promoter (MMP1-1607, rs:1799750) is particularly associated with increased MMP-1 mRNA transcriptional activity^29,32^. While previous studies suggest that genetic variations in MMP-2 and MMP-3 genes account for increased risk to periapical lesion formation^26^, the possible association of MMP-1 genetic variants was not investigated.

In addition to the influence of host genetic background, it is also important to consider that different cytokines may also account for the variance of MMP-1 levels in periapical tissues^2^. Indeed, strong and persistent microbial and inflammatory stimuli were described to override the genetic control of MMP-1 expression in periodontitis context^29^. Accordingly , pro-inflammatory cytokines are described to directly increase the levels of MMP-1, while anti-inflammatory cytokines are supposed to counteract such effect and to downregulate MMPs expression^8,14^. Interestingly, a marked dichotomy between pro- and anti-inflammatory cytokines clusters is associated with periapical activity/inactivity status^3^, suggesting that such mediators could in fact determine lesions activity via the regulation of MMPs expression^3^.

Therefore, in the present study we investigated the possible association of MMP1-1607 SNP with periapical lesions development risk, and simultaneously analyzed the impact of such polymorphism and host response mediators (TNF, IL-21, IL17, IFN-g, IL-10, FOXp3, IL-9, and IL-4) in the modulation of MMP-1 mRNA transcription in human chronic periapical granulomas.

## MATERIAL AND METHODS

### Subjects and samples

This study had institutional review board approval of Bauru School of Dentistry, University of São Paulo. Patients and control subjects were selected as previously described^3^. Patients presenting periapical lesions were referred to endodontic surgery after conventional root canal treatment failure; periapical lesions diagnosis was performed as previously described^25^ based on histopathological and radiographic analysis, being periapical lesions characterized radiographically as rarefaction lesions with the disappearance of the periodontal ligament space and discontinuity of the lamina dura. Treatment failure was defined as the presence of periradicular radiolucency that did not resolve, persisting as before acceptable endodontic treatment (i.e., having all canals instrumented and obturated, with no voids in the obturation mass, the apical terminus of the obturation at 1/1.5 mm from the radiographic apex), or that increased in size with evidences of continuous bone resorption)^2^. Periapical granulomas (N=111) were collected from patients (N=111, aged 19-59 years; 51 females and 59 males) during periapical surgery and divided in two roughly similar fragments and stored in both formalin (for routine histological examination performed after hematoxylin-eosin staining) and RNAlater solution (Ambion - Thermo Fisher Scientific Inc.; Waltham, Massachusetts, USA) (for molecular analysis). Test samples were limited to granulomas, histopathologically defined by the presence of capillaries, inflammatory cells, macrophages, and without the presence of an epithelial lining. Periapical cysts, where cavities were further developed and lined by stratified squamous epithelium, and partially epithelized lesions (epithelized granulomas) were excluded from the study. Periapical lesions were also categorized into putative active and inactive, based in the molecular profile of RANKL/OPG mRNA expression, as previously described^24^. Patients’ epithelial buccal cells were sampled from inner cheek buccal mucosa scrapping after a mouthwash with 3% glucose for genotyping purposes.

The control group (N=214) (patients aged 18-52 years; 103 females and 111 males) was comprised of subjects without a clinical history of pulpal or periapical pathology and were free from periapical lesions. Epithelial buccal cells of control subjects were sampled from inner cheek buccal mucosa scrapping after a mouthwash with 3% glucose for genotyping purposes. A subgroup of control subjects (N=26) (patients aged 18-35 years; 13 females and 11 males) was comprised of subjects scheduled for premolar extraction due to orthodontic purposes; in addition to epithelial buccal cells sampling, during the surgery periapical tissue samples were isolated, stored in RNA later solution and used as control specimens.

Patients and controls with medical conditions requiring the use of systemic modifiers of bone metabolism or other assisted drug therapy (i.e*.* systemic antibiotics, anti-inflammatory, hormonal therapy) during the last six months before the study were excluded. Patients and controls with preexisting conditions, such as periodontal disease and pregnant or lactating women, were also excluded.

### DNA extraction and analysis of MMP1-1607 SNP (rs1799750)

DNA was extracted from epithelial buccal cells with sequential phenol/chloroform solution as previously described^11^. Extracted DNA was used for genotyping. DNA integrity was checked and the allelic discrimination of MMP1-1607 SNP (rs:1799750) variants was performed in 3 μL reactions using Taqman (Thermo Fisher Scientific Inc.; Waltham, Massachusetts, USA) chemistry as previously described^21^. For reaction quality control, a sample of known genotype was included in the plate and a no DNA template sample was included as negative control. Only genotypes with an automatic call rate >95% were considered, error rate was <3%. Samples that failed to provide a genotype were repeated in additional reactions; genotyping was performed blinded to group status, as previously described^7^.

### RNA extraction and RealTime-PCR

In brief, total RNA was extracted from samples by using the RNeasy kit (Qiagen Inc, Valencia, California, USA) according to the manufacturers’ instructions. The integrity of RNA samples was checked by analyzing 1 μg of total RNA on 2100 Bioanalyzer (Agilent Technologies, Santa Clara, California, USA) according to the manufacturers’ instructions. After RNA extraction, complementary DNA was synthesized by using 3 μg of RNA through a reverse transcription reaction using QuantiTectRT kit (Qiagen Inc, Valencia, California, USA). All cytokines/Th markers (TNF-α, IFN-γ, IL-17A, IL-21, IL-10, IL-4, IL-9, FOXp3) mRNA levels were measured by means of RealTimePCR using TaqMan chemistry (Thermo Fisher Scientific Inc.; Waltham, Massachusetts, USA) in a Viia7 instrument (Thermo Fisher Scientific Inc.; Waltham, Massachusetts, USA) using inventoried optimized primers/probes sets (Invitrogen, Carlsbad, CA), with basic reaction conditions (40 cycles) 95°C (10’), 94°C (1’), 56°C (1’), and 72°C (2’). The analysis of RANKL and OPG mRNA levels were also determined in all the lesions (also by RealTimePCR using TaqMan chemistry), in order to categorize each sample in putative active and inactive lesions based on the RANKL/OPG ratio as previously described^24^. The results are depicted as the relative level of gene expression; calculated in reference to internal controls GAPDH and β-actin expression in each sample using the 2^-^Ct method.

### Data analysis

The Shapiro-Wilk test was performed to test the distribution of all test groups prior to comparative analysis; p>0.05 was considered indicative of normal distribution. Chi-squared test with 1 degree of freedom was performed to test the Hardy-Weinberg equilibrium in the allele frequencies of the study population. The differences in the demographic data for the study population and genotype and allele distribution among groups were tested by Fisher’s exact test and the equality of proportions test. Correlations between the MMP1-1607 genotype and the mRNA expression of pro inflammatory (MMP-1, TNF-α, IL-17A, IL-21, and IFN) and anti-inflammatory (FoxP3, IL-4, IL-9, IL-10) biomarkers was assayed by the Pearson’s product-moment correlation coefficient. The interrelation between the MMP1-1607 genotype and the MMP-1 mRNA levels was assayed by ordered logistic regression. The relative contribution of the explanatory variables MMP1-1607 genotype and the expression levels of the pro inflammatory (TNF-α, IL-17A, IL-21, and IFN) and anti-inflammatory (FoxP3, IL-4, IL-9, IL-10) biomarkers over the expression of MMP1 was evaluated by ordinal least squares (OLS) regression. A p-value <0.05 was considered statistically significant. All tests were performed in Stata14 (StataCorp LP College Station, Texas, USA) or GraphPad Prism 6.05 (GraphPad Software, Inc, San Diego, California, USA).

## RESULTS

### MMP1 -1607 SNP frequency analysis

The subject sample included in this study was similarly composed by male and female subjects (Table 1). The frequency of MMP1-1607 SNP genotypes and alleles in control group was similar to that previously reported for the Brazilian population^24^ and the Chi-square test for the distribution of genotypes in patients and controls rendered a p-value of 0.469 and 0.243, respectively, which was compatible with the Hardy-Weinberg equilibrium. When the patients presenting periapical lesions were compared with the controls, it was observed that the frequency of MMP1-1607 1G/2G was higher in the patients group (p=0.0110, OR=2.143, Cl=1.209 to 3.799), as well the combined mutant genotypes 1G/2G+2G/2G was more frequent in the patients (p=0.0326, OR=1.764, Cl=1.047 to 2.971) group than in controls (Table 1). When the frequency of the alleles was compared between the patients and controls groups, no differences were found. The frequency of the MMP1-1607 SNP genotypes and alleles was also compared within patient’s presenting active and inactive periapical lesions; however, no significant variations were found in the frequencies (Table 2).

**Table 1:** Frequencies of MMP1-1607 SNP in control subjects and patients presenting periapical lesions

	Control (N=214)	Lesions (N=111)	p value
N and gender distribution	103 f / 111 m	87 f / 91 m	0.8398*
MMP1-1607 genotypes	C (N=214)	L (N=111)	p value*
1G/1G	75 (35.04)	26 (23.42)	
1G/2G	70 (32.71)	52 (46.84)	p=0.0110 OR=2.143 Cl=1.209 to 3.799
2G/2G	69 (32.24)	33 (29.72)	p= 0.3542 OR=1.380 Cl= 0.7501 to 2.537
1G/2G + 2G/2G	139 (64.95)	85 (76.57)	p= 0.0326 OR=1.764 Cl=1.047 to 2.971
MMP1-1607 alleles	C (n=428)	CP (n=222)	
1G	220 (51.40)	104 (46.84)	
2G	208 (48.59)	118 (53.15)	p= 0.2830 OR=1.200 Cl=0.8673 to 1.661

* chi-square test, **unpaired t test; OR: odds ratio; Cl: confidence interval

**Table 2:** Frequencies of MMP1-1607 SNP in patients presenting active and inactive periapical lesions

	Active (N=40)	Inactive (N=71)	p value
MMP1-1607 genotypes	A (N=40)	I (N=71)	p value*
1G/1G	9 (22.50)	17 (23.94)	
1G/2G	17 (42.50)	35 (49.29)	p=1.0000 OR= 1.090 Cl= 0.4032 to 2.947
2G/2G	14 (35.00)	19 (26.76)	p=0.5988 OR=0.7185 Cl=0.2482 to 2.080
1G/2G + 2G/2G	31 (77.50)	54 (76.05)	p=1.0000 OR=0.9222 Cl= 0.3671 to 2.316
MMP1-1607 alleles	C (n=83)	CP (n=142)	
1G	38 (45.78)	69 (48.59)	
2G	45 (54.21)	73 (51.40	p=0.7821 OR=0.8934 Cl= 0.5190 to 1.538

* chi-square test, **unpaired t test; OR: odds ratio; Cl: confidence interval

### Association between MMP1-1607 with MMP-1 mRNA expression

**Figure 1:**
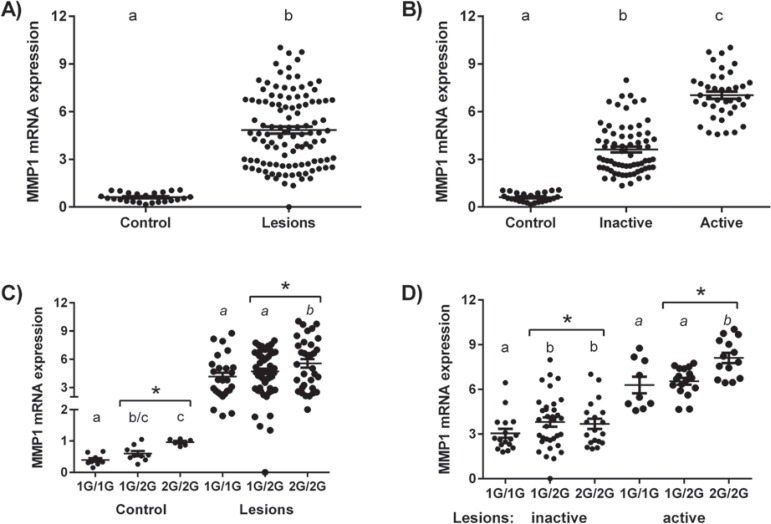
MMP1-1607 SNP and its association with MMP-1 mRNA levels in periapical tissues. Total RNA was extracted from healthy periodontal ligament (control, N=26) and periapical granulomas (lesions, N=111) and levels of MMP-1 mRNA were measured quantitatively by RealTimePCR using TaqMan chemistry; the results are presented as expression of the individual mRNAs (with normalization to beta-actin using the Ct method). A) MMP-1 expression in healthy periapical tissues and periapical lesions; B) MMP-1 expression in healthy periapical tissues and periapical lesions categorized into active or inactive based on the profile of RANKL/OPG expression; C) MMP-1 expression in healthy periodontal ligament and periapical lesions patients according to their genotype for MMP1-1607, determined by RealTimePCR using Taqman chemistry; D) MMP-1 expression in periapical lesions categorized into active or inactive based on the profile of RANKL/OPG expression according to patients genotype for MMP1-1607, determined by RealTimePCR using Taqman chemistry. Different letters (a, b, c) represent statistically significant differences within the groups (p<0.05; unpaired t-test or One-way ANOVA, Bonferroni post-test); * represents statistically significant differences between 1G/1G and 1G/2G+2G/2G groups (p<0.05; unpaired t-test)

Our data initially showed a weak MMP-1 expression in healthy periapical tissues sampled from control subjects, while a significantly stronger expression was evidenced in the periapical lesions harvested from patients (Figure 1). In addition, our data demonstrate that MMP-1 mRNA levels were significantly higher in active than in inactive lesions (Figure 1). When analyzing the possible association between MMP-1 mRNA levels and the MMP1-1607 SNP genotypes in control group, we found that polymorphic allele 2G (1G/2G+2G/2G genotypes) carriers presented a significantly higher MMP-1 mRNA expression when compared with the 1G/1G genotype group (Figure 1). Similar results were observed within the periapical lesions, where the carriers of polymorphic allele 2G (1G/2G+2G/2G genotypes) carriers presented a significantly higher MMP-1 mRNA expression than 1G/1G genotype carriers (Figure 1). When the periapical lesions were evaluated after stratification by lesion activity/inactivity status, it was observed that the expression of MMP-1 was higher in 1G/2G+2G/2G versus 1G/1G genotypes in both subgroups (Figure 1). Additionally, the ordered logistic regression pointed to a significant influence of the MMP1-1607 SNP over the mRNA MMP-1 expression levels, demonstrated by a likelihood ratio (LR) chi-squared of 6.09 (probability of > chi-squared=0.0136).

### Association between MMP-1 and mRNA expression of cytokines

**Figure 2:**
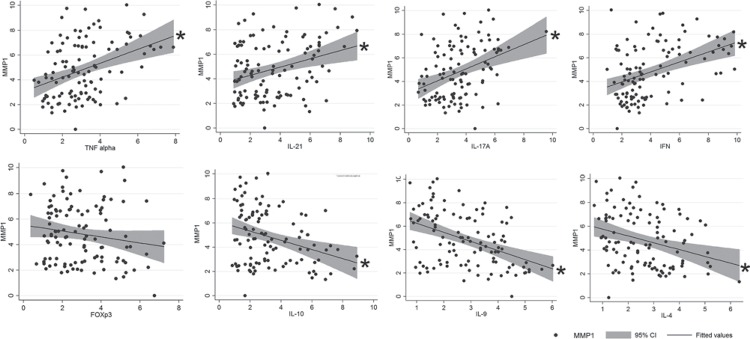
Cytokines, T helper markers and their association with MMP-1 mRNA levels in periapical lesions. Total RNA was extracted from periapical granulomas (N=111) and levels of MMP-1, TNF-a, IL-21, IL17A, IFNg, FOXp3, IL-10, IL-9, and IL-4 mRNA were measured quantitatively by RealTimePCR using TaqMan chemistry (with normalization to beta-actin using the Ct method). The graphs depict the linear correlation of MMP-1 and the other markers expression levels periapical lesions, the fitted values and the 95% confidence interval; * represents statistically significant correlation between the factors, the correlation coefficients between MMP-1 expression and cytokines’ expression levels are described in the results section

Cytokines and T helper markers previously associated with periapical lesions activity and inactivity clusters were correlated with MMP-1 mRNA transcripts to identify possible associations. The correlation coefficients between MMP-1 expression and expression levels of cytokines were: TNF-α 0.3812, p<0.0001; IL-21 0.3141, p=0.0008; IL-17A 0.3681, p=0.0001; IFN-γ 0.4206 p<0.0001; FOXp3 -0.1581, p=0.09; IL-10 -0.3166, p-=0.0008; IL-9 -0.4376, p<0.0001; IL-4 -3041, p=0.0012. Our results demonstrated that the cytokines associated with lesion activity clusters, namely TNF-α, IL-21, IL-17A, and IFN-g were positively correlated with MMP-1 levels (Figure 2), while IL-10, IL-9, and IL-22, cytokines associated with lesions inactivity, presented negative correlations with MMP-1 transcripts (Figure 2). No significant correlations were observed between the levels of MMP-1 and regulatory T cell marker FOXp3 (Figure 2).

### Regression analysis

Since MMP1-1607 and multiple cytokines were found to be associated with MMP-1 mRNA levels in periapical lesions, we subsequently performed a OLS regression analysis to model the relative contribution of the MMP1-1607 SNP and each biomarker to the overall MMP-1 regulation (Table 3). The first model integrated the genetic polymorphism and the expression levels of the pro-inflammatory cytokines TNF-α, IL-21, IL-17A, and IFN-γ as explanatory variables. The R-square was 0.4044, p<0.0001 (adjusted R-square 0.3757), with the parameter estimate coefficients 0.5507 for MMP1-1607 polymorphism (p=0.025); 0.3897 for IL-17A (p=0.001); 0.3261 for IFN-γ (p=0.3261); 0.2629 for TNF-α (p=0.031), and 0.1889 for IL-21 (p=0.028). The second model integrated the genetic polymorphism and the expression level of the anti-inflammatory biomarkers FoxP3, IL-4, IL-9, and IL-10 as explanatory variables. The R-square was 0.3253, p<0.0001 (adjusted R-square 0.2928), with the parameter estimate coefficients -0.6067 for IL-9 (p<0.0001); 0.5181 for MMP1-1607 polymorphism (p>0.05); -0.4059 for IL-4 (p=0.011); -0.2552 for IL-10 (p=0.033); and -0.0377 for FoxP3 (p>0.05). For each model, the R-square represented the proportion of the variance of MMP-1 expression attributed to the variation of the explanatory variables (adjusted by the number of variables in the case of adjusted R-square). The significant coefficients represent the value of the regression equation for predicting the MMP-1 expression from the explanatory variable, holding all other variables constant. For example, the IL-9 coefficient of -0.6067 (p<0.0001) predicts that for every unit increase in IL-9 we expect a -0.6067 decrease in MMP-1 expression, holding all other variables constant (Table 3 and Figure 3).

**Table 3:** OLS regression coefficients, standard error and p-value of 2-tailed t-static (null hypothesis that the coefficient is 0) with cytokines associated with lesions inactivity and activity. The coefficient represents the amount of change in the dependent variable that a unit change in each of the explanatory variables produces, holding all other variables constant. p>/t/ represents the two-tailed p-values used in testing the null hypothesis that the respective coefficient is equal to zero (has no effect in the dependent variable)

	Coefficient	Std. error	P>/t/
FoxP3	-0.0377	0.1424	0.791
IL-10	-0.2552	0.1184	0.033
IL-9	-0.6067	0.1501	<0.0001
IL-4	-0.4059	0.1564	0.011
TNF-α	0.2629	0.1201	0.031
IL-21	0.1889	0.085	0.028
IL-17A	0.3897	0.1088	0.001
IFNγ	0.3261	0.0808	<0.0001

## DISCUSSION

The increased expression of proteolytic enzymes, such as MMP-1, is supposed to contribute to the onset and progression of periapical lesions by promoting the degradation of soft and mineralized tissues surrounding the tooth apex. In this context, the identification of the factors that determine the control of MMP-1 expression in periapical environment may comprise important information to predict lesions development or to develop therapeutic strategies to limit lesion’s progression.

Our results initially demonstrate that MMP-1 mRNA levels were increased in periapical lesions when compared with healthy periapical tissues, in accordance with previous reports^1,31^. In addition, active periapical lesions present a significantly higher MMP-1 expression than inactive lesions, reinforcing the hypothesis that MMP-1 in fact contribute to lesions maintenance or progression. Accordingly, previous studies demonstrate that active lesions present a reduced expression of healing markers when compared with inactive ones as well as distinct cytokine profiles^15,23^. It is important to highlight that the overall MMP-1 expression in the periapical lesions, despite its activity status, was evidently variable. Therefore, our next step was to investigate if a genetic variation in the MMP-1 gene promoter, namely MMP1-1607 SNP, previously described as a regulator of MMP-1 expression levels^29^, could account for the MMP-1 mRNA modulation in periapical lesions as well as for an increased risk for lesions development.

Regarding the genetic association point of view, our results demonstrate a higher frequency of the 1G/2G genotype, as well as of the combined 1G/2G+2G/2G polymorphic genotypes, in the subjects presenting periapical lesions than in the control group. Therefore, such genotypes add a significant risk for the individual susceptibility to develop periapical lesions. Interestingly, a previous study demonstrate the association of MMP-2 and MMP-3 variants with periapical lesion formation in individuals with untreated deep carious lesions^26^, suggesting that individuals genetically prone to increased production of proteolytic enzymes may experience an increased risk for periapical lesions. It is important to consider that the increased risk observed in this study may be underestimated, since the control population does not comprise individuals strictly exposed to the major risk factors for periapical lesions such as untreated deep carious lesions^9,26^. While the selection of different control populations may in fact impact the power and odds of genetic studies^16^, the identification of a given risk factor, such as MMP1-1607 SNP, in a case-control setting that theoretically minimizes the changes of association, reinforces that this factor in fact comprises a risk element^9,15,26^. However, no differences were observed in the frequency of the MMP1-1607 genotypes and alleles in the patients presenting active or inactive lesions; however, the limited power of such fragmented analysis due to the dilution of the experimental sample within two subgroups do not allow definitive conclusions in this specific analysis.

After the identification of positive genetic association of MMP1-1607 with periapical lesion’s risk, we next evaluated from the functional viewpoint if the MMP-1 genetic variants were in fact associated with MMP-1 transcripts levels in the lesions. In fact, our results demonstrate that the MMP1-1607 2G allele was associated with increased MMP-1 mRNA expression in both healthy periapical tissues and in the periapical lesions. Accordingly, such polymorphism was previously described to affect gene transcription *in vitro*, since the 2G allele, together with an adjacent adenosine, creates a core binding site (5´-GGA-3´) for transcription factors immediately adjacent to an AP-1 site, causing a significant increase in the transcription activity^22^. In addition, the ordered logistic regression analysis demonstrate that the MMP1-1607 SNP is a significant predictor of MMP-1 levels in periapical tissues (LR chi-squared of 6.09, probability of > chi-squared=0.0136). Unfortunately, the ordered nature of the genetic data precludes the computation of an R-squared capable of predicting the relative effect of the polymorphism on the MMP-1 expression variation, nevertheless the sequential iterative computation of OLS regressions including and excluding the polymorphism as explanatory variable in various models (including incrementally all other explanatory variables) points to a contribution ranging between 2 and 5% (data not shown). This seemingly weak effect must be interpreted carefully, since OLS models are a simplification of complex phenomena, particularly in the context of biological processes. More important than the computed predictive value is the fact that the genetic polymorphism demonstrated a significant predictive effect on the overwhelming majority of the regressions computed.

It is compulsory to consider that numerous SNPs are in fact supposed to contribute to a given outcome with a significant but relatively small biological effect. Interestingly, in a previous study our group described the association between MMP1-1607 and the MMP-1 mRNA levels in healthy periodontal tissues, while in periodontal lesions the presence of microbial and inflammatory stimuli seems to overcome the genetic predisposition to higher MMP-1^29^. In this context, since periapical lesions are plenty of inflammatory signals, we next investigated if pro-inflammatory cytokines previously associated with periapical lesions activity^3^ could contribute to the modulation of MMP-1 expression levels. Our results demonstrate that the cytokines associated with lesion activity clusters, namely TNF-α, IL-21, IL-17A, and IFN-g were positively correlated with MMP-1 levels upon individual linear regression analyzes. Accordingly, all this cytokines were previously described as positive regulators of MMP-1 expression in different models^8^. When the OLS regression analysis was applied to consider the occurrence of the SNP and the level of expression of cytokines simultaneously, IL-17A and IFN-γ were ranked as the major determinants of MMP-1 levels in periapical lesions, with coefficients of 0.3897 and 0.3261, respectively. Interestingly, high levels of IL-17A and IFN-γ were observed in the cytokine cluster associated with the highest degree of periapical lesions activity^3^, but were also individually implicated with lesions’ activity in the clusters ranked in 2 and 3 positions^3^, reinforcing that their involvement in lesions’ progression may in fact involve the upregulation of MMP-1. The OLS regression analysis also demonstrated that TNF-α and IL-21 significantly account for MMP-1 regulation with coefficients of 0.2629 and 0.1889, respectively. In accordance, both TNF-α and IL-21 were described as components of the top 3 ranked cytokine clusters associated with periapical lesions activity^3^. It is mandatory to mention that the OLS regression computed simultaneously the explanatory effect of the cytokines and the MMP1-1607 SNP over the overall regulation of MMP-1 levels in periapical lesions.

On the other hand, the cytokines prevalent in clusters associated with lesions inactivity, namely IL-10, IL-9, and IL-4^3^, were found to be inversely correlated with MMP-1 levels in periapical lesions upon individual linear regression analyzes. Similarly, the OLS regression analysis demonstrated that IL-10, IL-9, and IL-4 significantly account for downregulating MMP-1 mRNA levels (coefficients -0.2552, -0.6067, and -0.4059, respectively). While some studies demonstrate that IL-10 can block MMPs production (especially MT1-MMP, MMP-1, and MMP-9) by tumoral cells^33^, there are no direct evidences of the direct inhibition of MMP-1 by IL-10, IL-9, or IL-4. However, it is possible to hypothesize that the negative correlation is derived from an indirect effect, since the anti-inflammatory properties of such cytokines, especially the most studied IL-10, result in the downregulation of the cytokines responsible for MMP-1 upregulation, such as TNF-α, IL-21, IL-17A, and IFN-γ^27^.

It is also interesting to explore the lack of correlation between FOXp3 and MMP-1 expression in both linear and OLS regression analysis. FOXp3 is the prototypic transcription factor of Tregs (regulatory T cells), a T cell subset with potent immunoregulatory properties, described to attenuate experimental periodontal and periapical lesions progression *in vivo* ^11,17^. In human periapical lesions, the expression of FOXp3 as well as its methylation levels are indicators of lesions inactivity status^2^. In active periapical lesions, increased FOXp3 methylation was associated with reduced FOXp3 mRNA expression, as well as reduced levels of IL-10, a characteristic immunoregulatory product of Tregs^5^. However, since FOXp3 is a regulator of Tregs biology, which in turn are supposed to modulate lesions’ inflammatory milieu via IL-10 (as well as via other immunoregulatory molecules such as TGF-b and CTLA-4), the existence of multiple elements in the immunoregulatory cascade between FOXp3 and MMP-1 (i.e., in a hypothetical oversimplified pathway, FOXp3 regulates the development of Tregs, which produce IL-10, which in turn can downregulate the levels of the inflammatory cytokines that directly control MMP-1 expression)^5^ may account for the lack of correlation between such factors. Indeed, while *in vivo* evidences link Tregs with the modulation of events coordinated by MMPs and TIMPs, such as tissue remodeling, degradation and fibrosis, the current evidences points to an indirect regulation of MMPs/TIMPs balance derived from cytokines produced and regulated by Tregs instead of a direct regulation^17^. Additionally, it is noteworthy that computed regression models fail to capture the subtle and intermingled regulatory interdependence between multiple explanatory variables and that the simultaneous inclusion of ordinal and continuous data in the OLS tends to underestimate the relative contribution of the continuous variables.

Taken together, our results demonstrate that multiple host factors seem to contribute to the local modulation of MMP-1 levels in periapical lesions. The MMP1-1607 SNP was demonstrated to be functional; being the 2G genotypes associated with increased MMP-1 mRNA levels as well as identified as a risk factor for periapical lesions development in the genotypic case-control association analysis. In addition, the cytokines TNF-α, IL-21, IL-17A, and IFN-γ were found to be associated with increased MMP-1 levels in periapical lesions, while IL-10, IL-9, or IL-22 presented an inverse association. Still, further cause-and-effect studies are required to dissect the intracellular networks involved in the regulation of MMP-1 levels upon multiple and divergent pro- and anti-inflammatory stimuli in different genetic backgrounds scenarios. Such studies may support (or discard) the application of MMP1-1607 SNP as biomarker for periapical lesions risk, as well to develop strategies to modulate MMP-1 levels in the clinical practice, and therefore contribute to improve the diagnosis and clinical management of these pathologies.

## CONCLUSION

The MMP1-1607 SNP was identified as a risk factor for periapical lesions development as well as to be associated with increased MMP-1 mRNA expression in periapical tissues and lesions. The expression of MMP-1 is also under the control of the pro- and anti-inflammatory milieu elements, being the cytokines TNF-α, IL-21, IL-17A, and IFN-g associated with increased MMP-1 levels in periapical lesions, while IL-10, IL-9, or IL-4 presented a negative correlation with MMP-1 expression.
